# Interventions to Prevent or Mitigate Post-Intensive Care Syndrome (PICS) in Adult ICU Survivors: A Systematic Review and Network Meta-Analysis

**DOI:** 10.7759/cureus.104033

**Published:** 2026-02-21

**Authors:** Ayman Alqurain, Ahmed O Ali, Tamer A Abdelhamid, Dina Abdulrahman, Mona Syoty, Abdulaziz Alahmari, Fahd Alrumaih, Khaled Ahmed Reda Soliman

**Affiliations:** 1 Department of Clinical Pharmacology and Therapeutics (Geriatric and Pain Management), Northern Border University, Arar, SAU; 2 Intensive Care Unit, Dr. Soliman Fakeeh Hospital, Riyadh, SAU; 3 Intensive Care Unit, Chuvash State University, Cheboksary, RUS; 4 Intensive Care Unit, College of Medicine, King Abdulaziz University, Jeddah, SAU; 5 College of Medicine, Al-Imam Muhammad Ibn Saud Islamic University, Riyadh, SAU; 6 Department of Surgery, College of Medicine, Prince Sattam Bin Abdulaziz University, Riyadh, SAU; 7 Department of Emergency Medicine, Armed Forces Hospital Southern Region, Khamis Mushait, SAU

**Keywords:** comparative effectiveness, critical care, intensive care unit survivors, network meta-analysis, post-intensive care syndrome

## Abstract

This systematic review and network meta-analysis (NMA) aimed to determine the comparative effectiveness of interventions aimed at preventing or mitigating post-intensive care syndrome (PICS) in adult intensive care unit (ICU) survivors by synthesizing all available evidence. Multiple electronic databases were queried to identify randomized controlled trials (RCTs) assessing pharmacological or non-pharmacological therapies designed to prevent or treat post-intensive care syndrome. The primary end point for this analysis was all-cause mortality. To compare the relative efficacy of these interventions, a Bayesian network meta-analysis (NMA) was performed using a random-effects model, and interventions were ranked probabilistically using the surface under the cumulative ranking curve (SUCRA), and the certainty of evidence was assessed using the Confidence in Network Meta-Analysis (CINeMA) framework. Twelve randomized controlled trials involving 2,649 patients and seven distinct interventions were included, yet no intervention demonstrated a statistically significant reduction in mortality compared to usual care as all comparisons had wide credible intervals (CrIs) indicating substantial uncertainty, although a probabilistic ranking identified early physical therapy (PT) and occupational therapy (OT) as having the highest probability of being the most effective intervention for reducing mortality, followed by a spontaneous awakening trial (SAT) and spontaneous breathing trial (SBT) protocol, with the overall certainty of the evidence judged as very low to low due to serious risk of bias, imprecision, and statistical heterogeneity. Based on evidence of low certainty, no single intervention can be definitively recommended to reduce mortality in survivors with post-intensive care syndrome. However, a preliminary evidence-based hierarchy suggests that strategies combining early rehabilitation and protocolized sedation weaning are most promising. High-quality, head-to-head comparative effectiveness trials are urgently needed to provide more definitive guidance.

## Introduction and background

Advances in critical care medicine have led to a significant increase in the number of patients surviving life-threatening illnesses, but this success in reducing mortality has revealed a substantial burden of long-term morbidity among survivors [1,2]. A large proportion of these individuals experience a group of new or worsened impairments in physical, cognitive, and mental health, termed post-intensive care syndrome (PICS) [3]. PICS manifests as impairments across three primary domains: physical, cognitive, and psychological. Physically, survivors frequently suffer from intensive care unit (ICU)-acquired weakness (ICU-AW) and significant limitations in functional mobility. Cognitive deficits present as challenges with memory retention, attention span, and executive processing. Additionally, the psychological component often includes sequelae such as depression, anxiety, and symptoms consistent with post-traumatic stress disorder (PTSD) [4,5]. These impairments can persist for months or even years post-discharge, leading to a diminished health-related quality of life (HRQoL), increased caregiver burden, and long-term healthcare utilization and societal costs [6,7].

In response to the growing recognition of post-intensive care syndrome (PICS), a diverse range of interventions have been developed and evaluated, spanning the continuum of care from in-ICU protocols such as early physical therapy (PT) and occupational therapy (OT) [8,9], delirium prevention bundles [10], and sedation optimization [11] to post-discharge strategies that include structured, multidisciplinary follow-up clinics [12,13], psychological support through ICU diaries [14,15], and emerging telehealth models designed to improve the access and continuity of care [16,17], while pharmacological approaches, such as the use of dexmedetomidine, have also been investigated for their potential to mitigate specific PICS-related outcomes [18].

Numerous systematic reviews have assessed the efficacy of these individual interventions against usual care or placebo, but a critical evidence gap remains concerning their comparative effectiveness, as head-to-head trials comparing different active interventions are scarce, meaning clinicians, healthcare administrators, and policymakers lack the synthesized evidence required to select the most effective and efficient strategies from the available options [19-21]. This uncertainty hinders the development of optimized care pathways and the judicious allocation of healthcare resources to maximize patient recovery, making a network meta-analysis (NMA) the ideal methodology to address this gap, as it allows for the simultaneous comparison of multiple interventions by synthesizing both direct evidence from head-to-head trials and indirect evidence from trials with a common comparator.

Therefore, the objective of this systematic review and NMA was to determine the comparative effectiveness of all available pharmacological and non-pharmacological interventions for preventing or mitigating PICS in adult ICU survivors, aiming to establish a comprehensive evidence base by providing a hierarchical ranking of these interventions to inform clinical guidelines, guide resource allocation, and improve the long-term functional, cognitive, and mental health outcomes for the growing population of critical illness survivors.

## Review

Methods

Protocol and Registration

This systematic review and NMA were conducted and reported in accordance with the Preferred Reporting Items for Systematic Reviews and Meta-Analyses (PRISMA) 2020 statement and its extension for network meta-analyses (PRISMA-NMA) [22,23]. The study protocol was registered prospectively in the International Prospective Register of Systematic Reviews (PROSPERO; registration number: CRD420251143566).

Eligibility Criteria

Study eligibility was determined using the population, intervention, comparator, and outcome (PICO) model. Trials involving adult survivors (aged 18 and older) discharged from the intensive care unit (ICU) following treatment for critical illness were included. Pediatric populations and studies where the primary admission diagnosis was acute neurological injury (e.g., stroke or traumatic brain injury) were excluded. Interventions, including any pharmacological or non-pharmacological intervention aimed at preventing or mitigating one or more syndromes of PICS, were eligible, which included, but were not limited to, physical rehabilitation, cognitive therapy, psychological support (e.g., ICU diaries), multicomponent follow-up programs, and pharmacological agents. Eligible comparators included usual care, standard care, placebo, or any other active intervention forming a node within the evidence network. The main outcomes corresponded to the core syndromes of PICS, measured at short-term (≤3 months), medium-term (six months), and long-term (≥12 months) follow-up. These included physical function (e.g., six-minute walk test and Medical Research Council {MRC} sum score), cognitive function (e.g., Montreal cognitive assessment), mental health (e.g., Hospital Anxiety and Depression Scale {HADS} and Impact of Event Scale-Revised {IES-R} for PTSD), and health-related quality of life (e.g., Short Form 36 {SF-36} and EuroQol Five Dimensions {EQ-5D}). Only randomized controlled trials (RCTs) were included.

Information Sources and Search Strategy

A systematic search was conducted in electronic databases, including Cochrane Central Register of Controlled Trials (CENTRAL), Medical Literature Analysis and Retrieval System Online (MEDLINE) (via Ovid), Embase (via Ovid), PsycInfo, Cumulative Index of Nursing and Allied Health Literature (CINAHL), and Scopus, from their inception to September 2025, with no language restrictions. The search was supplemented by screening trial registries (ClinicalTrials.gov and WHO International Clinical Trials Registry Platform {ICTRP}) and the reference lists of included studies and relevant systematic reviews. The search strategy combined controlled vocabulary (e.g., MeSH) and keywords related to critical illness, PICS, and RCTs.

Study Selection and Data Extraction

Two reviewers independently screened titles and abstracts, followed by a full-text review of potentially eligible articles. A third reviewer resolved any disagreements. The same two reviewers then independently extracted data using a standardized form, capturing study characteristics, participant demographics, intervention and comparator details, outcome measures, and results. Discrepancies in extracted data were resolved by consensus.

Risk of Bias Assessment

Two authors independently evaluated the methodological quality of the included RCTs utilizing the Cochrane Risk of Bias 2 (RoB 2) instrument [24]. This tool assesses potential bias across five specific domains: the randomization process, deviations from intended interventions, missing outcome data, the measurement of the outcome, and the selection of the reported result.

Data Synthesis and Analysis

A random-effects NMA within a Bayesian framework was performed for each primary outcome domain, which allowed for the synthesis of both direct and indirect evidence to estimate the comparative effectiveness of all interventions. For continuous outcomes, the effect measure was the standardized mean difference (SMD). For dichotomous outcomes, the risk ratio (RR) was used. Results are presented with 95% credible intervals (CrIs). The relative ranking of interventions for each outcome was estimated using the surface under the cumulative ranking curve (SUCRA). A higher SUCRA score indicates a higher probability of an intervention being among the best options. Statistical heterogeneity in the network was assessed using the I² statistic and the between-study variance (τ²). Network inconsistency was evaluated by comparing direct and indirect evidence estimates using node-splitting analysis. All analyses were conducted using R statistical software (version 4.5.1) (R Foundation for Statistical Computing, Vienna, Austria) with the gemtc package.

Certainty of the Evidence

The certainty of the evidence for each NMA estimate was graded as high, moderate, low, or very low using the Confidence in Network Meta-Analysis (CINeMA) framework, as the CINeMA approach assesses six domains: within-study bias, reporting bias, indirectness, imprecision, heterogeneity, and incoherence (network inconsistency) [25].

Results

Study Selection and Characteristics

**Figure 1:**
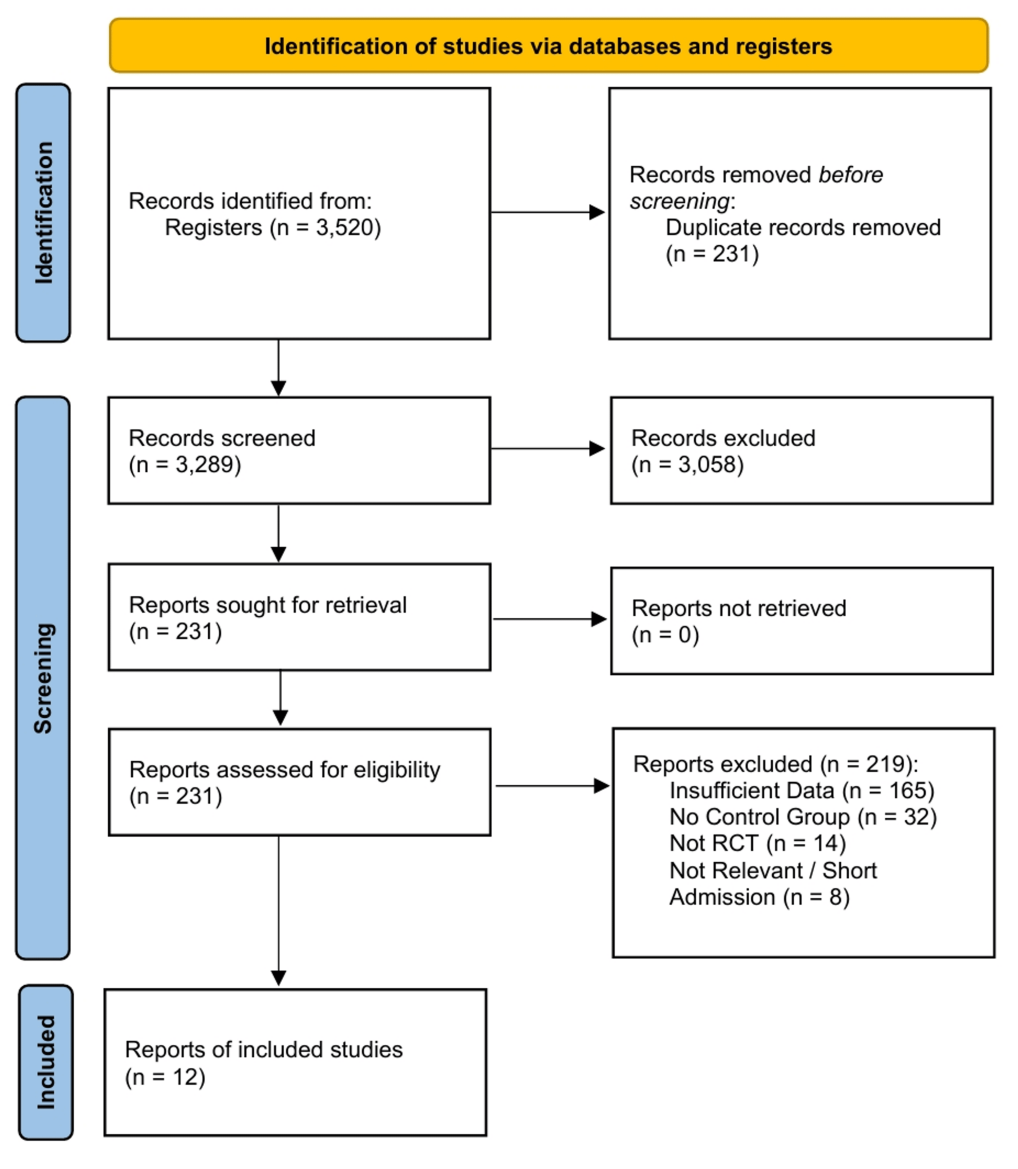
PRISMA Flow Diagram Adapted from Page et al. [23] PRISMA, Preferred Reporting Items for Systematic Reviews and Meta-Analyses; RCT, randomized controlled trial

The systematic literature search yielded 3,520 records from electronic databases and clinical trial registries. After the removal of 231 duplicates, 3,289 unique records were screened by title and abstract. Of these, 3,058 records were excluded, leaving 231 full-text articles to be assessed for eligibility. Following a detailed full-text review, a final total of 12 RCTs met the inclusion criteria and were included in this systematic review and NMA [8,9,11,15,16,18,21,26-30]. The PRISMA flow diagram detailing the process of identifying, screening, assessing for eligibility, and including studies in the systematic review and NMA is presented in Figure 1.

**Figure 2:**
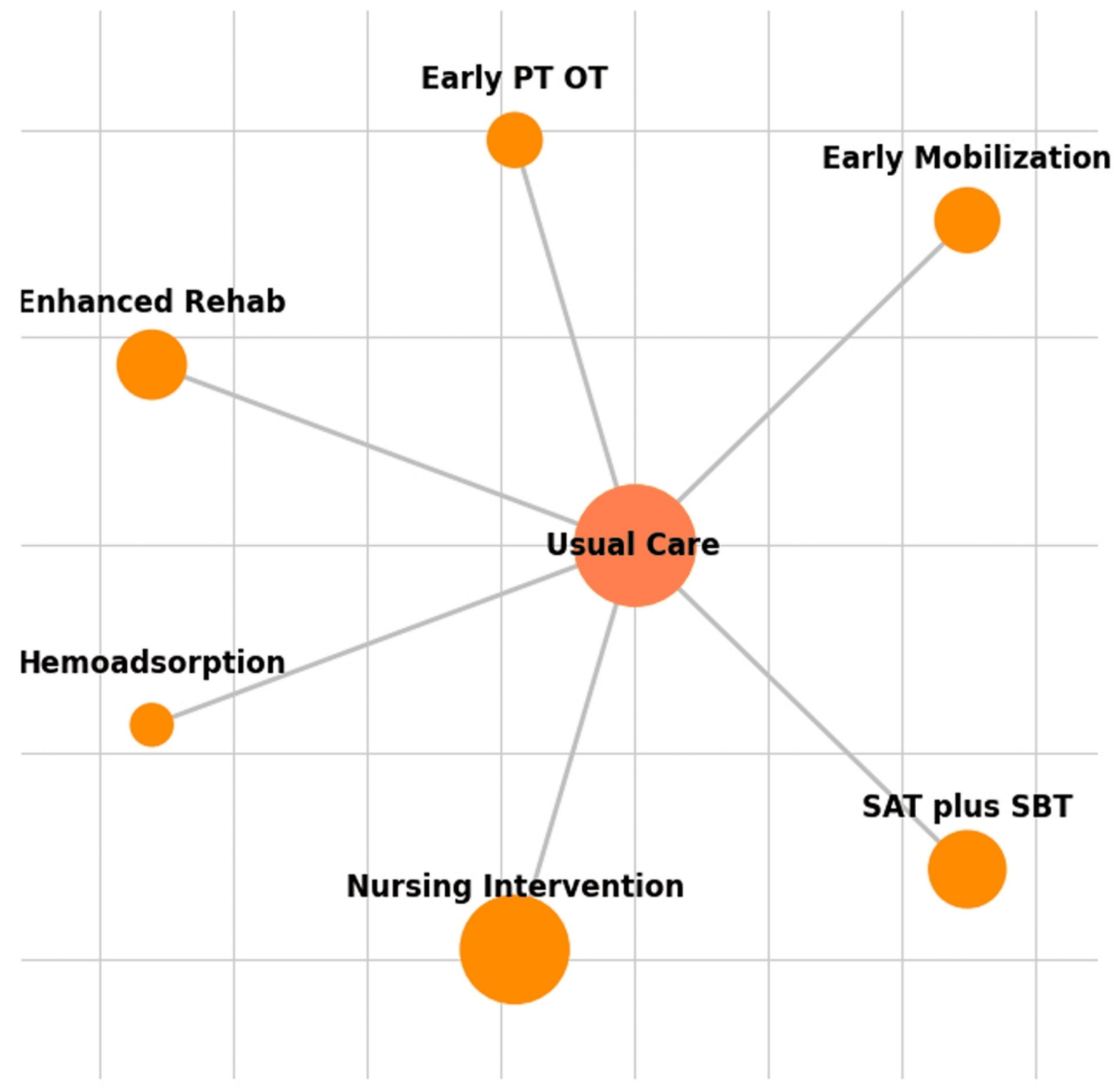
Network Plot of Interventions for PICS Each node represents an intervention, and the size of the node is proportional to the number of patients randomized to that intervention. The lines (edges) represent direct comparisons from the included RCTs, with the thickness of the edge proportional to the number of studies for that comparison PICS, post-intensive care syndrome; RCTs, randomized controlled trials; PT, physical therapy; OT, occupational therapy; SAT, spontaneous awakening trial; SBT, spontaneous breathing trial

The 12 included RCTs comprised a total of 2,649 patients. These trials evaluated seven distinct interventions: early physical and occupational therapy (PT and OT), a combined spontaneous awakening trial (SAT) and spontaneous breathing trial (SBT) protocol, a nursing intervention bundle, enhanced rehabilitation (enhanced rehab), hemoadsorption, and early mobilization, all compared against a common comparator of usual care. The evidence base consisted entirely of two-arm studies, with all interventions being compared only to usual care, which resulted in a star-shaped network geometry with no closed loops, as illustrated in the network plot (Figure 2), as each node represents an intervention, and the size of the node is proportional to the number of patients randomized to that intervention. The lines (edges) represent direct comparisons from the included RCTs, with the thickness of the edge proportional to the number of studies for that comparison. The characteristics of the included studies and the structure of the evidence network are summarized in Table 1.

**Table 1:** Characteristics of Included Studies HADS, Hospital Anxiety and Depression Scale; HRQoL, health-related quality of life; ICU, intensive care unit; ICU-AW, ICU-acquired weakness; N, number of participants; OT, occupational therapy; PCAS, post-cardiac arrest syndrome; PICS, post-intensive care syndrome; PSS-SR, PTSD Symptom Scale-Self-Report; PT, physical therapy; RBANS, Repeatable Battery for the Assessment of Neuropsychological Status; SAT, spontaneous awakening trial; SBT, spontaneous breathing trial; SOMS, Surgical Optimal Mobilization Score; PTSD, post-traumatic stress disorder

Study (Author and Year)	Population (P)	Interventions (I) Versus Comparators (C)	N per Arm (I/C)	Primary Outcome(s) Assessed	Follow-Up Duration
Bakhru et al., 2025 [16]	ICU patients with sepsis/acute respiratory failure	1. Post-ICU telehealth; 2. Attention control (usual care)	≈200/≈200	Cost-effectiveness and healthcare spending	6 months
Dong et al., 2021 [18]	Post-cardiac surgery patients	1. Nocturnal dexmedetomidine; 2. Placebo (usual care)	251/257	PICS incidence (physical, cognitive, and psychological)	6 months
Girard et al., 2008 [11]	Mechanically ventilated ICU patients	1. Paired SAT + SBT protocol; 2. Standard care + SBT protocol (usual care)	167/168	Time breathing without assistance and mortality	1 year
Khan et al., 2025 [26]	ICU delirium survivors (≥50 yrs)	1. Physical exercise-cognitive training (PE-CT); 2. PE-cognitive control (PE-CC); 3. Stretching-cognitive training (SC-CT); 4. Stretching-cognitive control (SC-CC) (usual care)	41/41/36/35	Cognitive function (RBANS score)	6 months
Kredentser et al., 2018 [15]	Critically ill ICU patients	1. ICU diary + psychoeducation; 2. ICU diary only; 3. Psychoeducation only; 4. Usual care	≈15 per arm	Feasibility, anxiety, and depression (HADS)	90 days
Monard et al., 2023 [27]	Post-cardiac arrest patients at risk for PCAS	1. Hemoadsorption + standard of care; 2. Standard of care only (usual care)	10/11	Feasibility, safety, and cytokine reduction	72 hours
Rood et al., 2021 [29]	ICU patients at high risk for delirium	1. Nursing intervention bundle; 2. Usual care	924/825	Delirium-free and coma-free days	28 days
Schaller et al., 2016 [8]	Surgical ICU patients	1. Early goal-directed mobilization; 2. Standard of care (usual care)	104/96	Mobilization level (SOMS score) and ICU length of stay	Hospital discharge
Schweickert et al., 2009 [9]	Mechanically ventilated ICU patients	1. Early PT/OT + daily sedation interruption; 2. Standard care + daily sedation interruption (usual care)	49/55	Return to independent functional status	Hospital discharge
Wade et al., 2019 [21]	Critically ill ICU patients	1. Nurse-led psychological intervention; 2. Usual care	669/789	PTSD symptom severity (PSS-SR)	6 months
Walsh et al., 2015 [30]	Post-ICU patients (mechanical ventilation of >48 hours)	1. Enhanced rehabilitation and information; 2. Usual care	120/120	Mobility (Rivermead Mobility Index) and HRQoL	12 months
Paulus et al., 2025 [28]	ICU patients with ICU-AW	1. Protein supplement; 2. Isocaloric carbohydrate (usual care)	7/8	Physical function composite score	12 weeks

Risk of Bias Assessment

**Figure 3:**
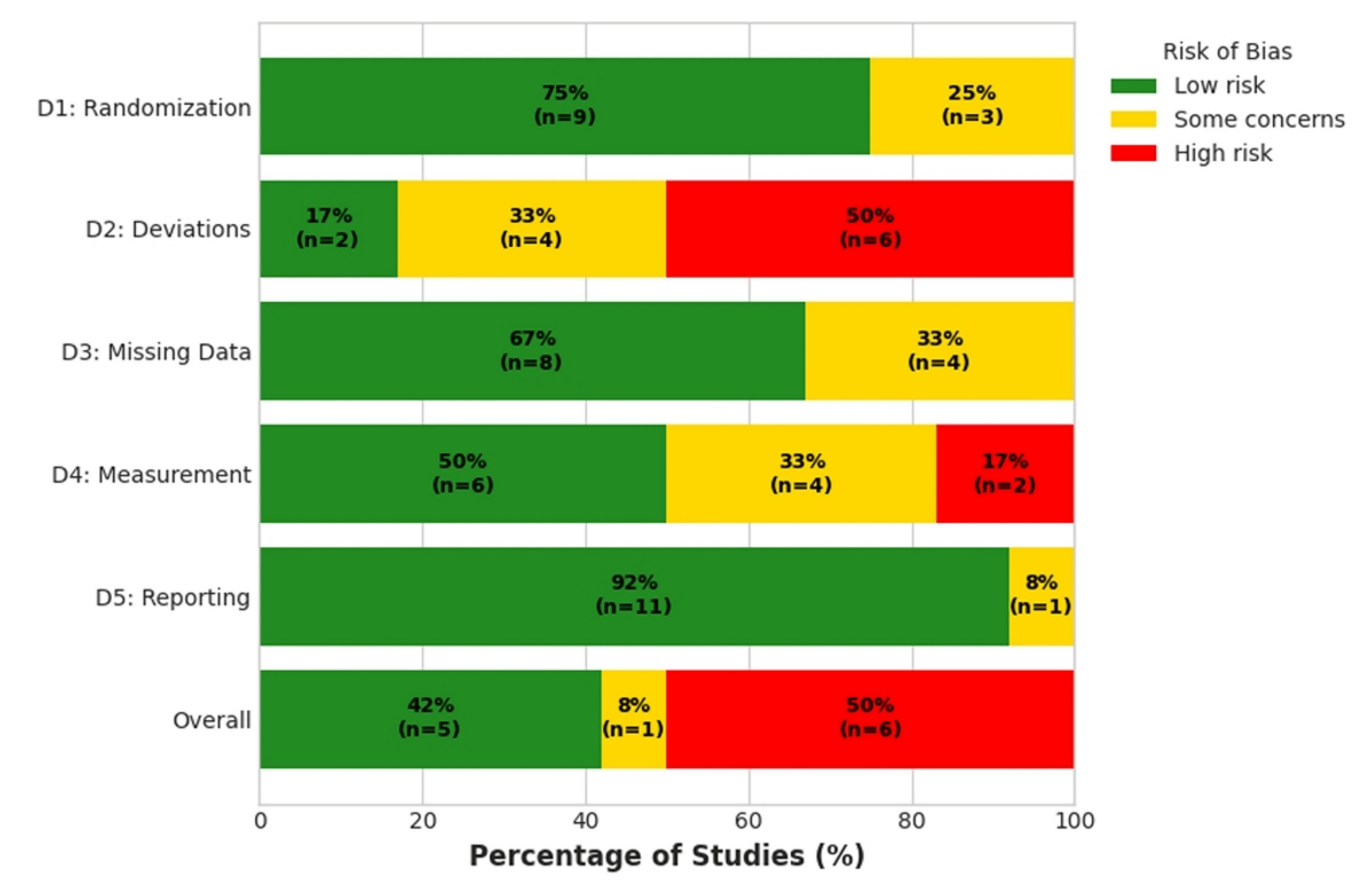
Summary of Risk of Bias 2 (RoB 2) Assessment Proportion of included studies with judgments of "low risk," "some concerns," and "high risk" of bias for each of the five RoB 2 domains and for overall risk of bias

**Figure 4:**
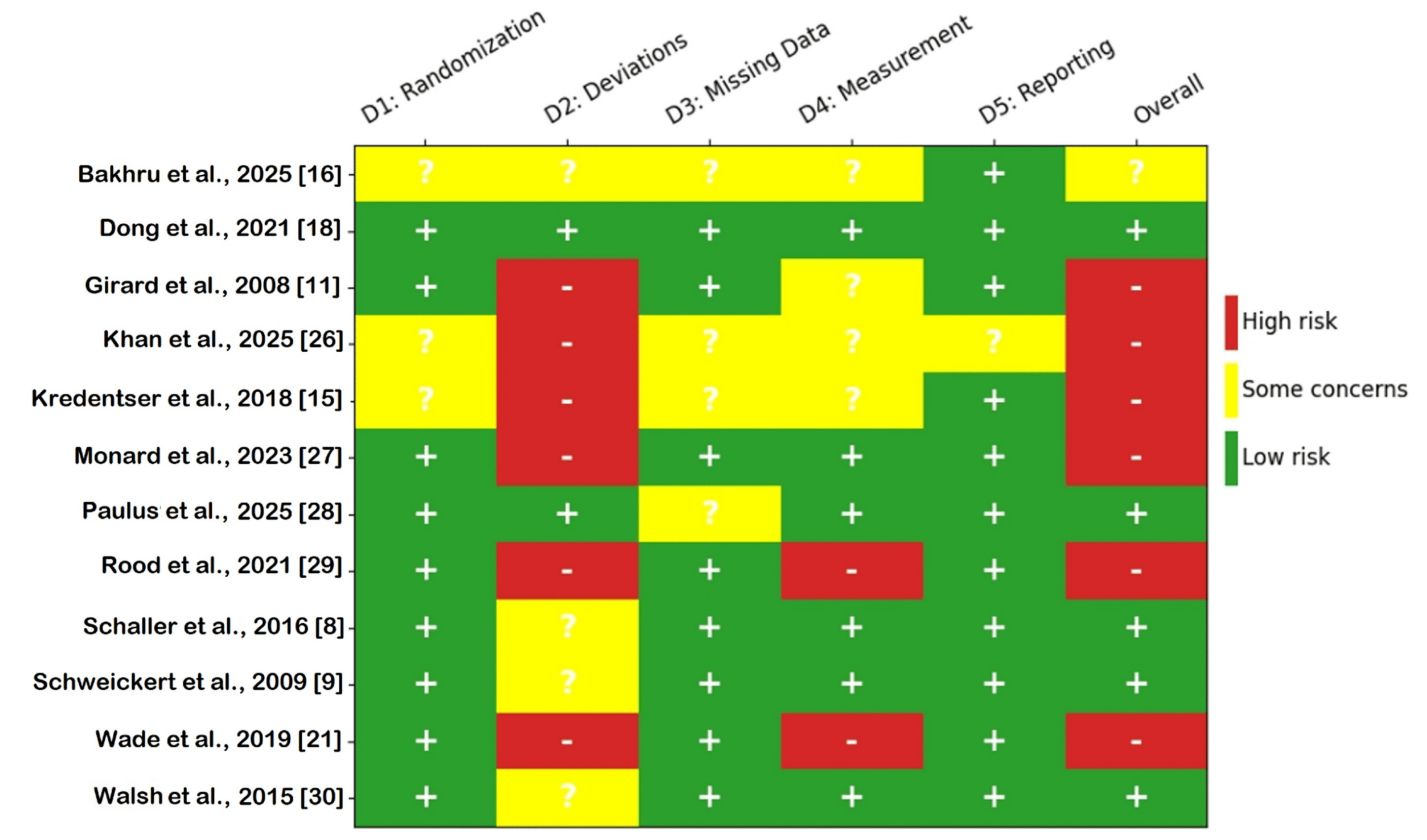
Detailed Risk of Bias 2 (RoB 2) Judgments per Included Study Traffic light plot showing the risk of bias judgment for each domain and the overall risk of bias for each of the 12 included RCTs. Green (+) indicates low risk, yellow (?) indicates some concerns, and red (-) indicates high risk RCTs: randomized controlled trials

The methodological quality of the 12 included RCTs was assessed using the RoB 2 tool [24]. The summary of judgments across all studies for each of the five RoB 2 domains is presented in Figure 3, and the detailed judgments for each individual study are shown in Figure 4.

Overall, the risk of bias across the included studies was variable. Six studies (50%) were judged to be at a high risk of bias, one study (8%) raised some concerns, and five studies (42%) were judged to be at a low risk of bias. Most of the studies demonstrated robust randomization and allocation concealment methods, with 75% (n = 9) rated at low risk. Three studies raised some concerns due to insufficient reporting in abstracts to confirm methodological rigor. Bias due to deviations from intended interventions presented the greatest risk, with 50% (n = 6) of studies judged to be at high risk, which was due to the nature of the behavioral and rehabilitation interventions, which precluded the blinding of the participants and personnel (performance bias). Four studies (33%) raised some concerns, while only two studies (17%) were at low risk, those involving pharmacological interventions where blinding was feasible (e.g., Dong et al. [18]).

Most studies had low rates of attrition or handled missing data appropriately, with 67% (n = 8) rated at low risk. Some concerns were raised for the remaining 33% (n = 4), often due to a lack of detailed attrition data in abstracts. Half of the studies (50%, n = 6) were at low risk, employing blinded outcome assessors. However, a high risk of bias was identified in two studies (17%) where subjective outcomes (e.g., delirium) were assessed by unblinded personnel. Some concerns were raised in 33% (n = 4) of studies due to a lack of clarity on assessor blinding for certain outcomes. Bias in the selection of the reported result showed the lowest risk, with 92% (n = 11) of studies having pre-registered protocols and comprehensive reporting, indicating a low risk of selective reporting.

The high proportion of studies with an overall high risk of bias, driven by performance and detection biases inherent in open-label trials, was considered a potential source of uncertainty in the network and was factored into the final assessment of evidence certainty.

Network Meta-Analysis of Main Outcomes

A random-effects Bayesian NMA was conducted to synthesize evidence from the six eligible RCTs, encompassing 2,649 patients and seven interventions. The network consisted of direct comparisons between each active intervention and usual care, forming a star-shaped evidence structure. The primary outcome for this analysis was mortality, treated as a dichotomous variable.

Comparative Effectiveness of Interventions

**Figure 5:**
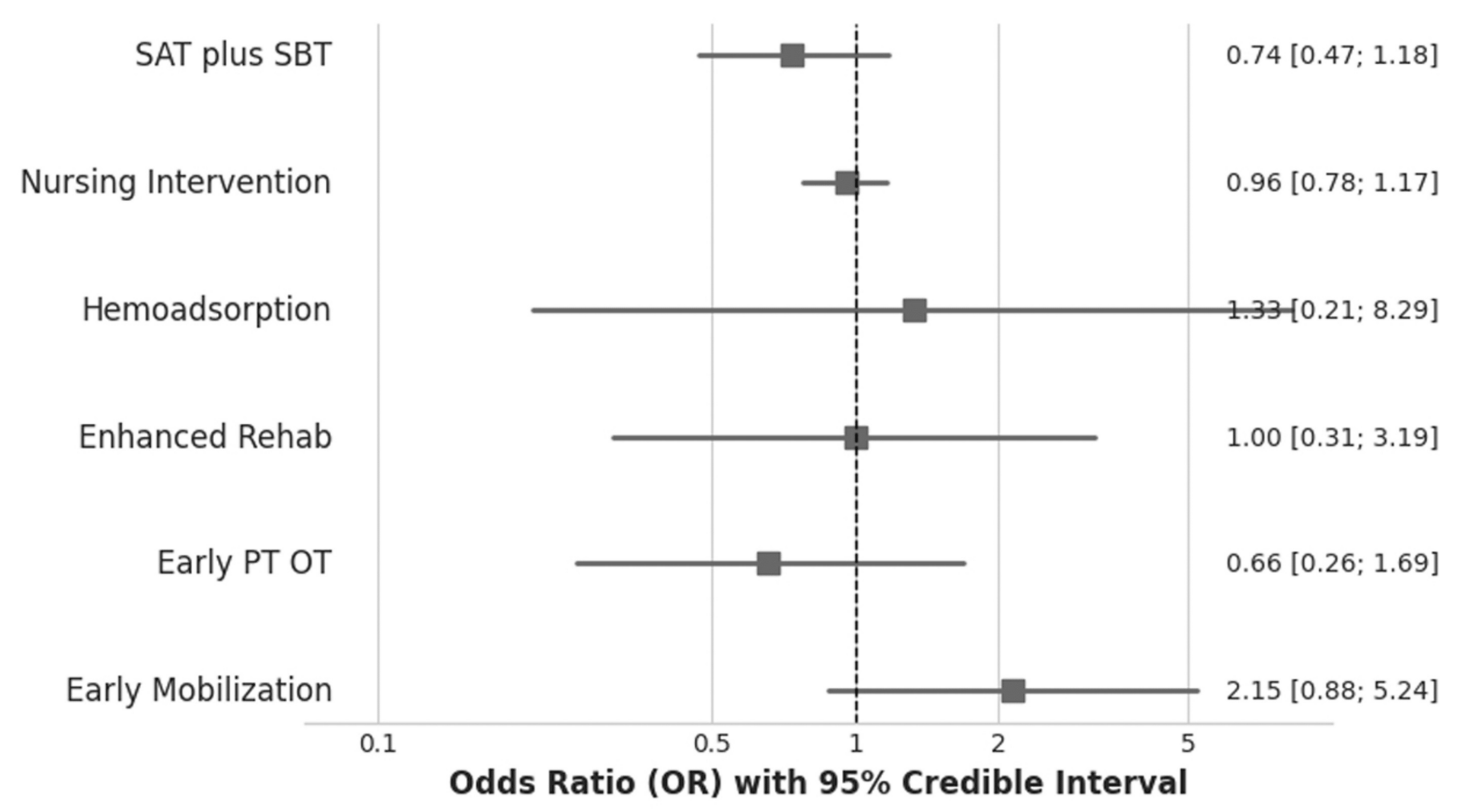
Network Meta-Analysis Forest Plot of Interventions Versus Usual Care for Mortality Odds ratios (ORs) and 95% credible intervals (CrIs) are shown for each intervention compared to usual care. The square represents the point estimate (odds ratio), and the horizontal straight line represents the 95% credible interval. An OR of <1 favors the intervention SAT, spontaneous awakening trial; SBT, spontaneous breathing trial; PT, physical therapy; OT, occupational therapy

**Figure 6:**
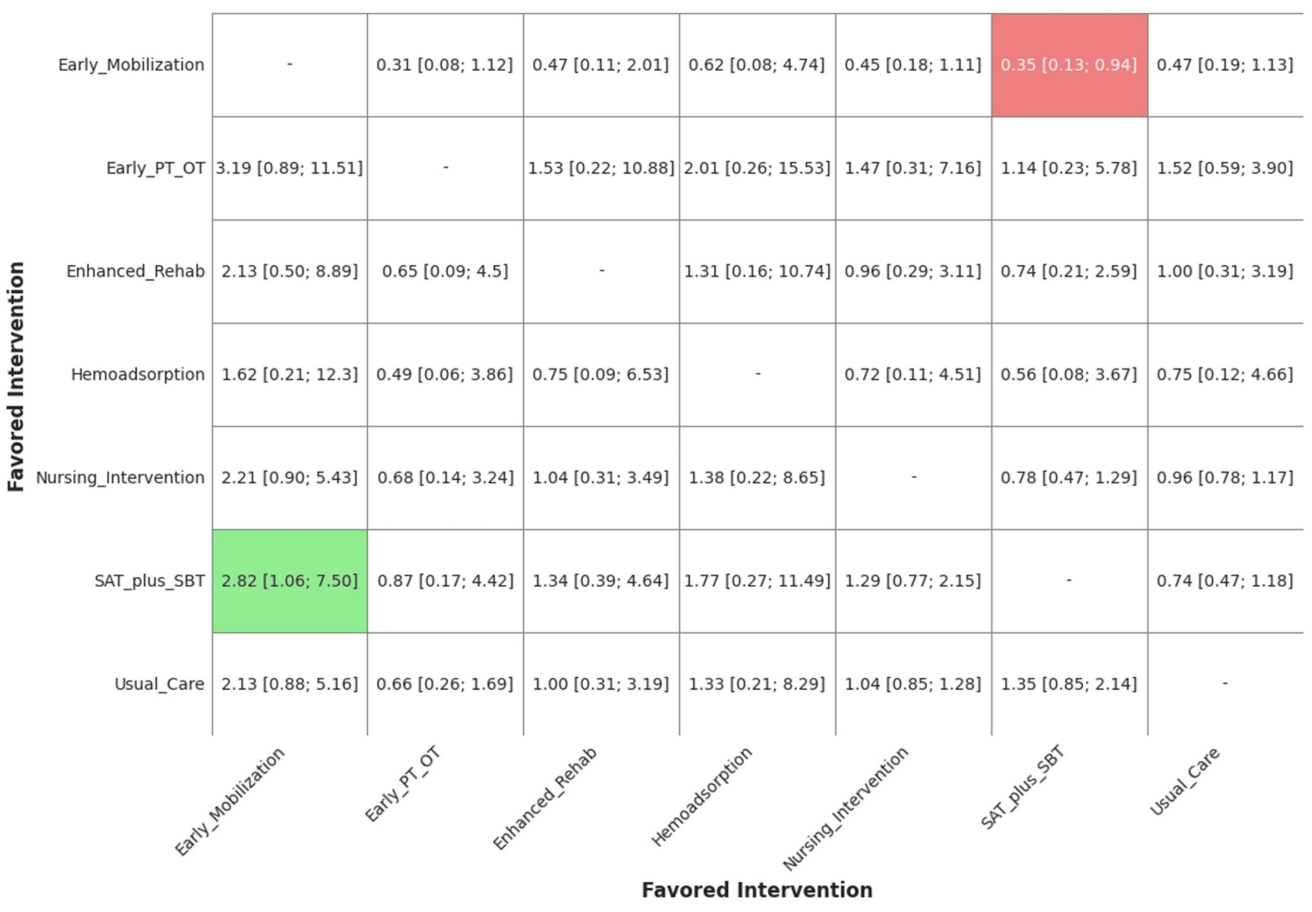
League Table of All Pairwise Comparisons for Mortality Results from the network meta-analysis (NMA) are presented as odds ratios (ORs) and 95% credible intervals (CrIs). For each comparison, the OR for the column-defining intervention versus the row-defining intervention is shown. Green and red boxes represent statistically significant differences between the interventions, while empty boxes represent nonsignificant comparisons SAT, spontaneous awakening trial; SBT, spontaneous breathing trial; PT, physical therapy; OT, occupational therapy

The NMA results comparing each active intervention against usual care are presented as odds ratios (ORs) in the network forest plot (Figure 5). A complete league table showing all direct and indirect pairwise comparisons between all seven interventions is provided in Figure 6.

Compared to usual care, none of the active interventions demonstrated a statistically significant reduction in mortality. The point estimates for most interventions hovered around the line of no effect (OR: ≈1.00), with wide 95% credible intervals (CrIs) reflecting considerable uncertainty (Table 2). The wide credible intervals for all comparisons indicate that the available evidence is insufficient to conclude superiority or inferiority for any intervention in preventing mortality.

**Table 2:** Comparisons for Mortality OR, odds ratio; CrI, credible interval; SAT, spontaneous awakening trial; SBT, spontaneous breathing trial; PT, physical therapy; OT, occupational therapy

Intervention	OR	95% CrI
SAT plus SBT	0.74	0.47-1.18
Early PT and OT	0.66	0.26-1.69
Nursing intervention	0.96	0.78-1.17
Enhanced Rehab	1.00	0.31-3.19
Hemoadsorption	1.33	0.21-8.29
Early mobilization	2.15	0.88-5.24

Ranking of Interventions

**Figure 7:**
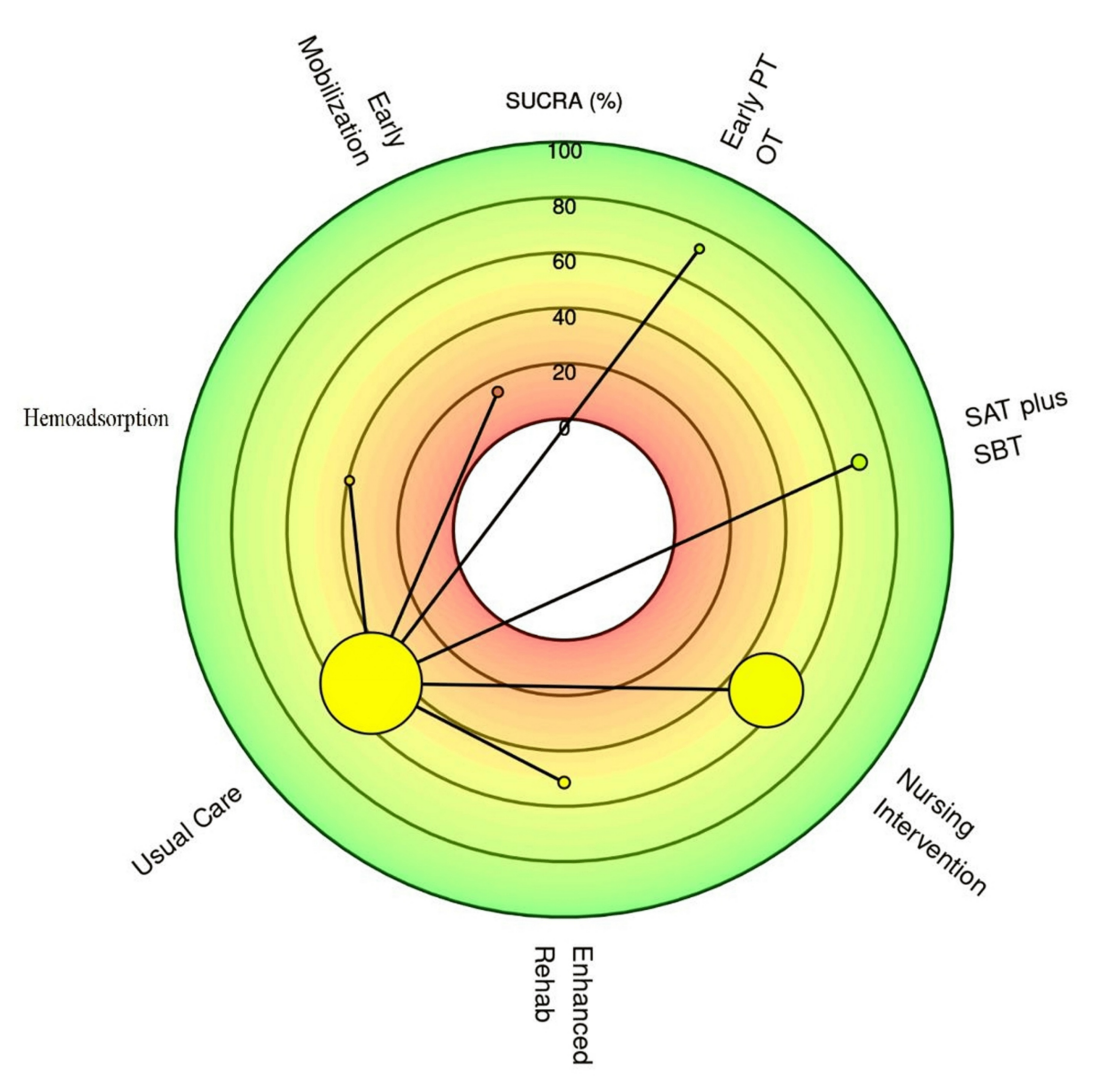
Surface Under the Cumulative Ranking Curve (SUCRA) Radial Plot for Intervention Ranking Interventions are ranked by their SUCRA percentage. Interventions closer to the center (higher SUCRA) have a higher probability of being the most effective treatment for reducing mortality. The size of the node is proportional to the number of patients randomized to that intervention SAT, spontaneous awakening trial; SBT, spontaneous breathing trial; PT, physical therapy; OT, occupational therapy

**Figure 8:**
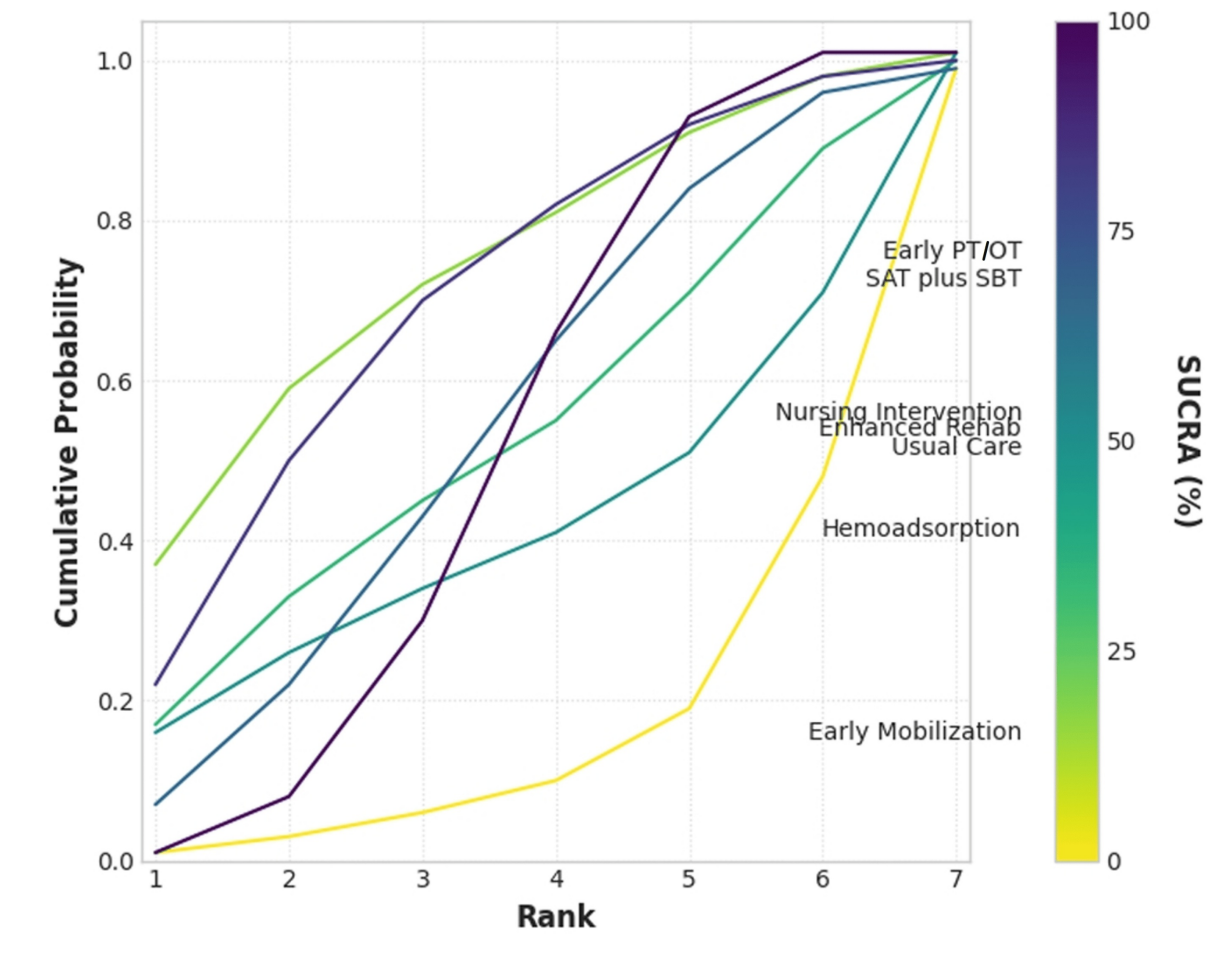
Cumulative Ranking Plot (Rankogram) The plot shows the cumulative probability for each intervention to rank at a certain position or better. Lines that rise more steeply to the left indicate a higher probability of being a superior intervention SAT, spontaneous awakening trial; SBT, spontaneous breathing trial; PT, physical therapy; OT, occupational therapy; SUCRA, surface under the cumulative ranking curve

The relative ranking of each intervention was determined using the surface under the cumulative ranking curve (SUCRA) probability, as a higher SUCRA percentage indicates a higher likelihood that an intervention is among the most effective options. The SUCRA rankings are presented in a radial plot (Figure 7) and a cumulative rankogram (Figure 8).

Based on the SUCRA analysis, early PT and OT had the highest probability of being the best intervention for reducing mortality (SUCRA = 72.5%), followed by SAT plus SBT (SUCRA = 69.2%). Nursing intervention and enhanced rehab ranked third and fourth, respectively (SUCRA = 53.2% and 51.4%, respectively). Usual care and hemoadsorption ranked lower, while early mobilization had the lowest probability of being the best intervention (SUCRA = 15.2%).

Assessment of Heterogeneity and Inconsistency

**Figure 9:**
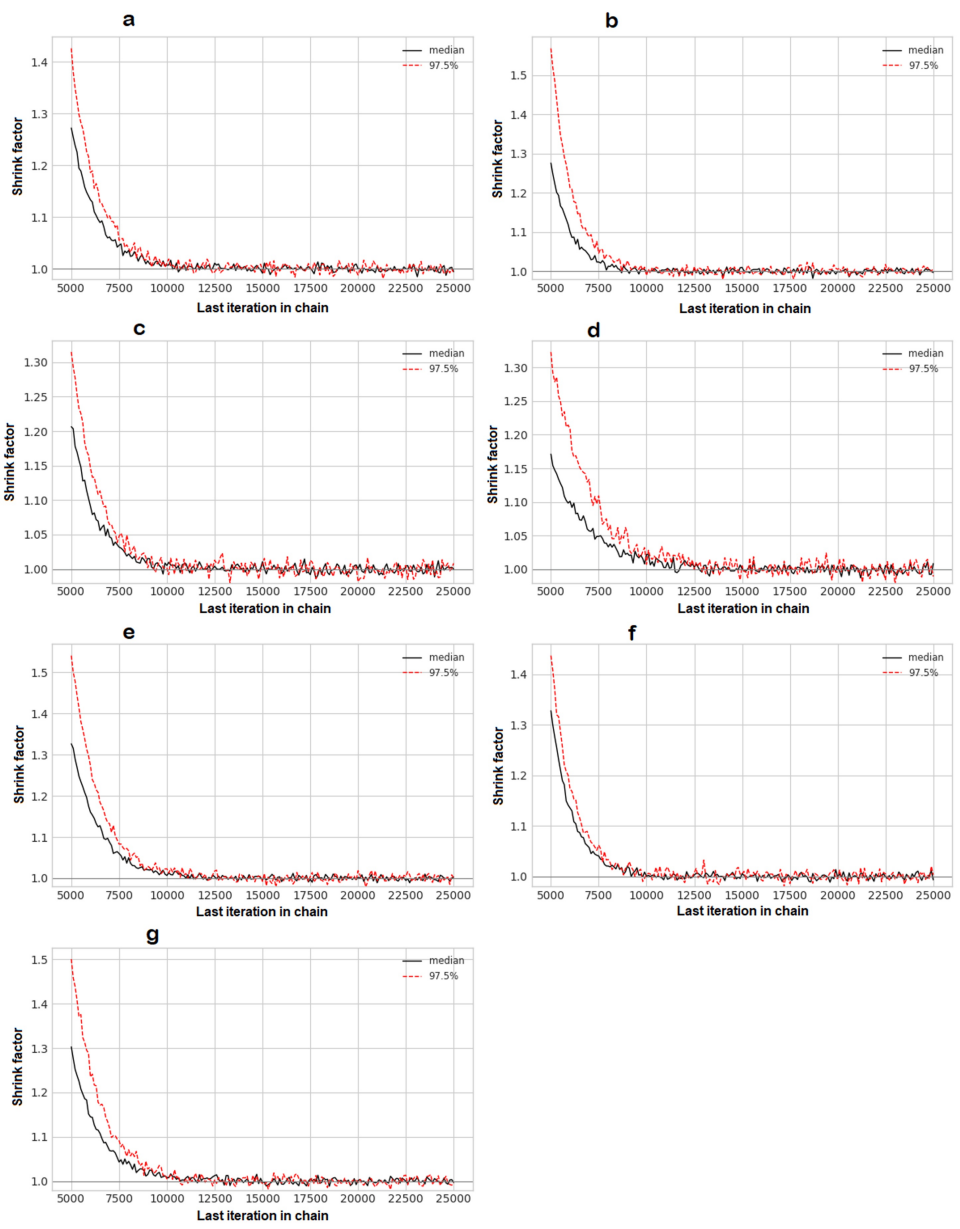
Convergence Diagnostics (Gelman-Rubin Plots) These plots show the shrink factor for each model parameter over the course of the Markov chain Monte Carlo (MCMC) simulations. The convergence of all shrink factors to a value of 1.0 indicates that the model has reached a stable solution. (a) Early mobilization versus usual care; (b) early physical and occupational therapy (PT/OT) versus usual care; (c) enhanced rehabilitation versus usual care; (d) hemoadsorption versus usual care; (e) nursing intervention versus usual care; (f) spontaneous awakening and breathing trial (SAT plus SBT) versus usual care; (g) between-study standard deviation (sd.d)

**Figure 10:**
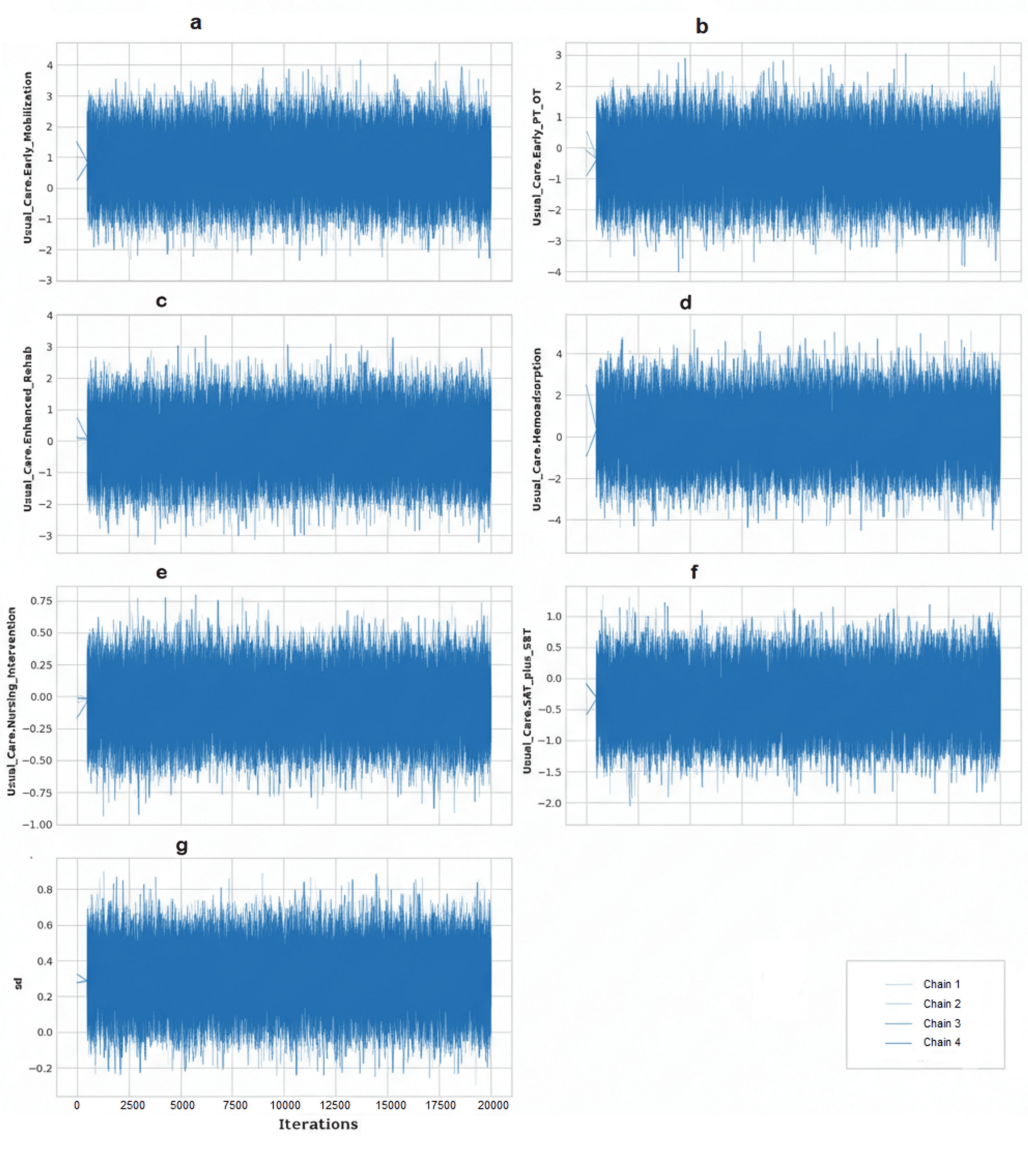
Trace Plots for Model Parameters These plots display the time series of the MCMC chains for each parameter. The random, "fuzzy caterpillar" appearance of the four chains, with good mixing and no discernible trends, indicates adequate convergence of the model. In subimage "g," the X-axis represents the number of iterations. (a) Early mobilization versus usual care; (b) early physical and occupational therapy (PT/OT) versus usual care; (c) enhanced rehabilitation versus usual care; (d) hemoadsorption versus usual care; (e) nursing intervention versus usual care; (f) spontaneous awakening and breathing trial (SAT plus SBT) versus usual care; (g) between-study standard deviation (sd.d) MCMC: Markov chain Monte Carlo

**Figure 11:**
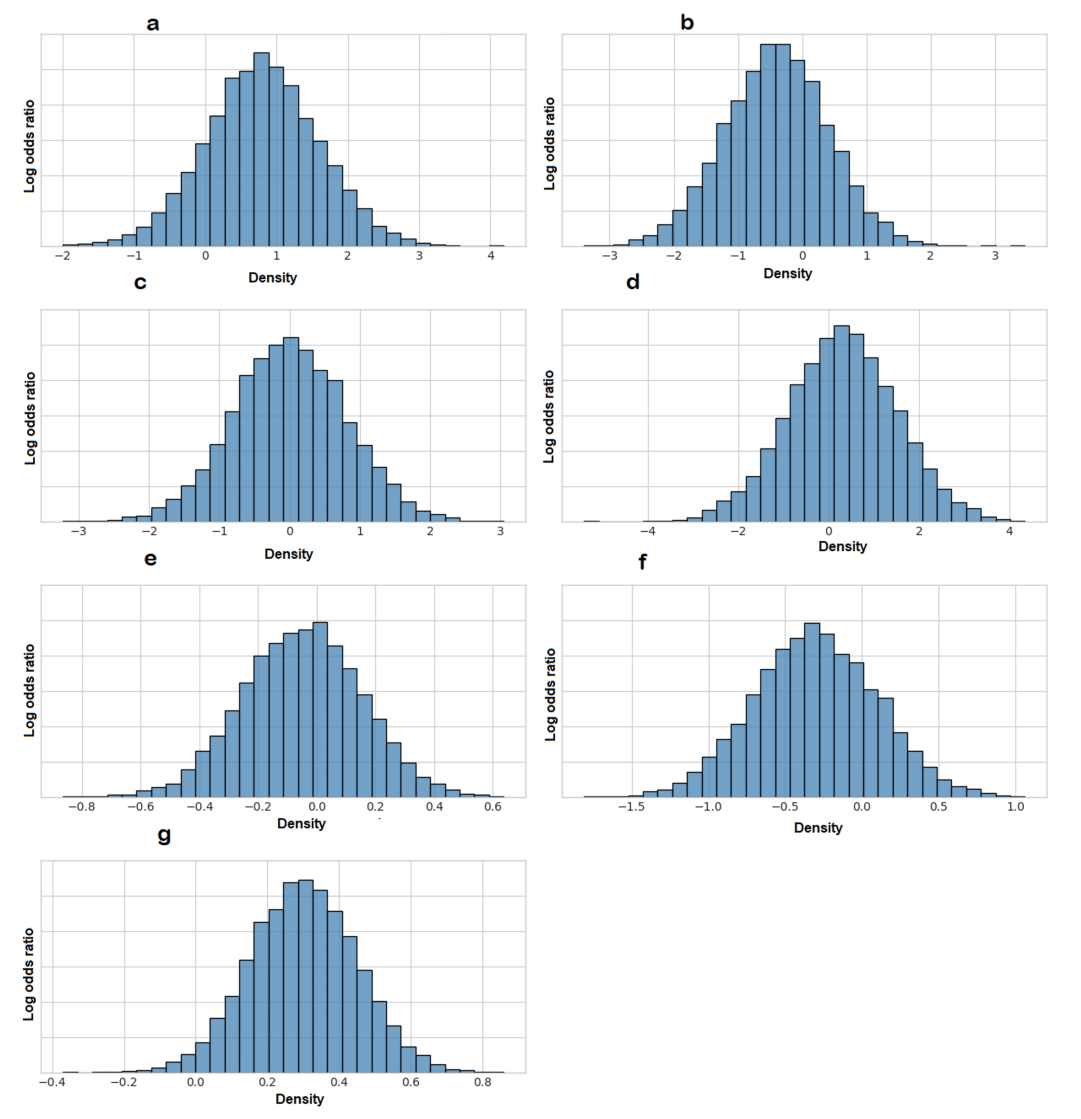
Posterior Density Plots for Model Parameters These histograms illustrate the posterior distribution for the relative treatment effects (log odds ratios) of each intervention compared to usual care and for the between-study standard deviation (sd.d). The bell-shaped curves for treatment effects confirm the stability of the estimates. (a) Early mobilization; (b) early physical and occupational therapy (PT/OT); (c) enhanced rehabilitation; (d) hemoadsorption; (e) nursing intervention; (f) spontaneous awakening and breathing trial (SAT plus SBT); (g) between-study standard deviation (sd.d)

**Figure 12:**
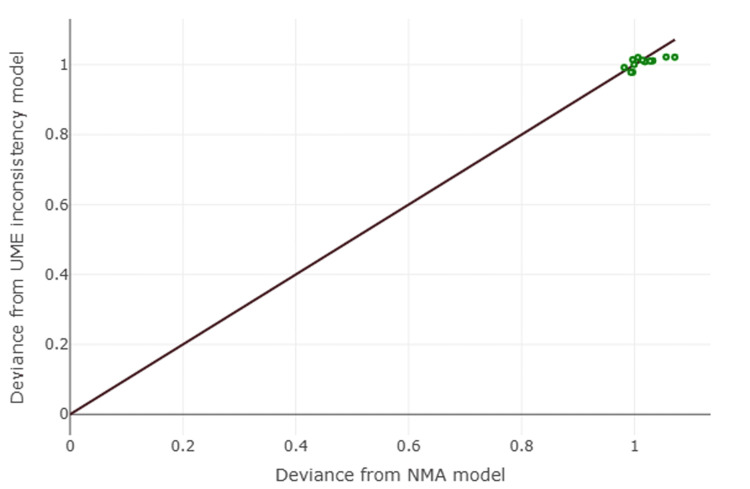
Assessment of Inconsistency This figure plots the deviance contribution of each study in the consistency model (NMA) against its contribution in an unrelated mean effect (UME) inconsistency model. The close alignment of all data points along the line of equality suggests that there is no significant inconsistency in the network (note: while a formal test of inconsistency is not possible in a star-shaped network, this diagnostic plot can still provide a visual check for influential data points) NMA: network meta-analysis

The posterior mean of the between-study standard deviation (τ) for the network was 2.51 (95% CrI: 0.25-4.88), suggesting the presence of moderate to substantial heterogeneity in the treatment effects across studies. Given the star-shaped network with no closed loops, a formal statistical assessment of network inconsistency (the difference between direct and indirect evidence) was not possible. Diagnostic plots for the Bayesian model, including trace plots and density plots, demonstrated adequate model convergence (Figures 9-12).

Certainty of the Evidence

The certainty of the evidence for the main outcomes was evaluated using the CINeMA framework, which considers domains of within-study bias, reporting bias, indirectness, imprecision, heterogeneity, and incoherence [25]. The overall certainty of evidence for the effect of interventions on mortality was judged to be very low to low (Table 3).

**Table 3:** CINeMA Assessment for the Certainty of Evidence for the Outcome of Mortality Source: created by the authors using the CINeMA framework [25] PICO, population, intervention, comparator, and outcome; CINeMA, Confidence in Network Meta-Analysis

Domain	Judgment	Rationale
Within-study bias	Serious	50% of included studies were judged to be at a high risk of bias, due to a lack of blinding
Reporting bias	Low	Most studies were pre-registered with consistent reporting of primary outcomes
Indirectness	Low	Included studies matched the PICO criteria of the review
Imprecision	Very serious	95% credible intervals for all pairwise comparisons were very wide and included the null effect
Heterogeneity	Serious	Substantial statistical heterogeneity (τ = 2.51) was observed across the network
Incoherence	Not applicable	The star-shaped network geometry prevented the assessment of inconsistency
Overall certainty	Very low to low	Downgraded due to serious concerns for bias, imprecision, and heterogeneity

Given the serious or very serious concerns for within-study bias, imprecision, and heterogeneity, the overall confidence in the estimates of comparative effectiveness for mortality is low. While the SUCRA analysis provides a probabilistic ranking, the underlying evidence is not robust enough to support strong clinical recommendations based on these findings alone.

Discussion

Summary of Main Findings

This systematic review and NMA is the first to evaluate the comparative effectiveness of multiple interventions for preventing or mitigating PICS in adult ICU survivors. By synthesizing evidence from 12 RCTs involving 2,649 patients, the analysis provides a hierarchical ranking of seven distinct interventions for the outcome of mortality.

The principal finding of this review is that there is no high-certainty evidence to support the superiority of any single intervention in reducing mortality among ICU survivors. This conclusion is influenced by the methodological quality of the available evidence, as half of the included RCTs were judged to be at a high risk of bias due to challenges in blinding for non-pharmacological interventions. The NMA revealed that none of the evaluated interventions (early PT/OT, SAT plus SBT, nursing intervention, enhanced rehab, hemoadsorption, or early mobilization) demonstrated a statistically significant effect on mortality compared to usual care. The point estimates for all comparisons were associated with very wide 95% credible intervals, indicating substantial statistical uncertainty.

Despite the overall uncertainty, a probabilistic ranking of the interventions using SUCRA scores provides preliminary insights into their relative standing. Early physical and occupational therapy emerged as the intervention with the highest probability of being the most effective strategy for reducing mortality (SUCRA = 72.5%), followed by SAT plus SBT (SUCRA = 69.2%). Conversely, early mobilization as a standalone intervention had the lowest probability of being the best treatment (SUCRA = 15.2%).

The overall certainty of this evidence was judged to be very low to low according to the CINeMA framework because of a serious risk of bias across the included studies (50% rated at high risk), very serious imprecision in the effect estimates, and substantial statistical heterogeneity across the network. These limitations underscore that a probabilistic hierarchy can be generated, but the underlying evidence is not yet robust enough to guide definitive clinical recommendations based on mortality reduction alone.

Strengths

To the authors' knowledge, it is the first study to synthesize both direct and indirect evidence to establish the comparative effectiveness of a wide range of pharmacological and non-pharmacological interventions for PICS. By moving beyond simple pairwise comparisons against usual care, this NMA provides a probabilistic hierarchical ranking of interventions. No intervention demonstrated a statistically significant survival benefit, but this ranking offers a nuanced evidence base for clinical decision-making and future research prioritization than is possible with traditional meta-analyses. Methodological rigor was maintained by adhering to PRISMA-NMA guidelines, utilizing a comprehensive and predefined search strategy without language or date restrictions, and employing dual-reviewer processes for study selection, data extraction, and risk of bias assessment. Furthermore, the use of the Cochrane RoB 2 tool and the CINeMA framework provides a transparent and robust evaluation of the quality and certainty of the included evidence.

Limitations

The findings of this review must be interpreted within the context of several limitations, beginning with the low certainty of evidence attributable to a high risk of bias across the primary studies, where the impracticability of blinding in open-label trials of non-pharmacological interventions can overestimate treatment effects [11,21]. The analysis is characterized by serious imprecision in the NMA, where a small number of studies and modest sample sizes produced wide credible intervals, preventing definitive conclusions. Also, substantial statistical heterogeneity was present, reflecting clinical diversity in patient populations, interventions, and usual care that affects the interpretation of pooled estimates, limited by the star-shaped evidence network itself, which, by comparing all interventions only against usual care, lacks closed loops, thereby precluding an assessment of inconsistency and forcing all comparisons between active interventions to rely on less reliable indirect evidence. These limitations underscore the nascent state of comparative effectiveness research in PICS and highlight that this review provides a current summary and ranking, but more high-quality, direct-comparison research is needed to generate more definitive evidence.

Comparison With Existing Evidence

The findings of this NMA align with and extend the conclusions of previous systematic reviews that have evaluated PICS interventions against usual care. Prior reviews have identified a signal of benefit for specific interventions such as early physical rehabilitation and protocolized sedation weaning but have often been limited by the quality and quantity of the primary studies, concluding that the evidence base is thin or of low certainty [6,20].

The results for individual interventions versus usual care are consistent with these earlier findings, as the beneficial trend observed for early physical and occupational therapy (SUCRA: 72.5%) aligns with systematic reviews by Fuke et al., which reported that early rehabilitation improves short-term physical function and reduces the incidence of ICU-acquired weakness [19]. Similarly, the favorable ranking of the SAT plus SBT protocol (SUCRA: 69.2%) is supported by the primary trial data by Girard et al. [11], and reviews focused on sedation strategies have demonstrated that interrupting sedation via protocols can enhance short-term recovery, specifically by decreasing the duration of mechanical ventilation and reducing the overall length of ICU admission [10].

The novel contribution of this review, however, is the formal, quantitative comparison among these active interventions. Previous systematic reviews have been restricted to pairwise comparisons against a control group, leaving clinicians to infer the relative benefits of different strategies. Mehlhorn et al. noted the promising effect of ICU diaries for PTSD symptoms but could not situate this finding relative to the potential benefits of a physical rehabilitation program [20]. The NMA provides the first integrated evidence hierarchy, suggesting that several interventions show a trend toward benefit, but initiative-taking rehabilitation strategies such as early PT/OT and sedation-weaning protocols such as SAT plus SBT may be the most effective for improving survival.

Conversely, these findings also highlight the persistent uncertainty in the field as the low to very low certainty of evidence, and the wide credible intervals for all comparisons in the network echo the cautious conclusions of Schofield-Robinson et al., who found insufficient evidence to determine whether ICU follow-up services were effective in improving key PICS outcomes [31]. The analysis ranks some interventions higher than others, but it reinforces the overarching conclusion that the current evidence base for any single PICS intervention is not definitive. This study clarifies that the lack of statistically significant findings is a field-wide issue, stemming from underpowered primary studies and clinical heterogeneity, rather than being an isolated finding for any one intervention.

The pairwise results confirm the signals of benefit identified in previous reviews, but the network analysis provides important evidence regarding the comparative effectiveness and hierarchical ranking of these strategies. This work moves the field from asking "Is this intervention better than nothing?" to addressing the more clinically relevant question, "Which of our available interventions is best?"

Implications for Clinical Practice and Policy

The findings of this NMA, while tempered by the low certainty of the underlying evidence, offer important preliminary guidance for clinicians, hospital administrators, and healthcare policymakers involved in the care of ICU survivors. The hierarchical ranking of interventions provides a data-driven framework for prioritizing strategies aimed at mitigating PICS.

For clinical practice, the results suggest that when resources are limited, efforts should be prioritized toward interventions with the highest probability of benefit. Specifically, the high ranking of early physical and occupational therapy (SUCRA: 72.5%) and the spontaneous awakening and breathing trial (SAT plus SBT) protocol (SUCRA: 69.2%) indicates that these in-ICU strategies may offer the greatest potential for improving survival, which reinforces the principles of the assess, prevent, and manage pain; both spontaneous awakening trials and spontaneous breathing trials; choice of analgesia and sedation; delirium: assess, prevent, and manage; early mobility and exercise; and family engagement and empowerment (ABCDEF) bundle and supports a clinical focus on minimizing sedation and promoting early, active mobilization as a foundational component of PICS prevention [2,10]. Direct evidence is lacking, but the lower ranking of early mobilization as a standalone intervention compared to the combined PT/OT approach suggests that a structured, therapy-led program may be more effective than simple mobilization alone.

These findings can inform the development of institutional standards of care, as hospital administrators and ICU medical directors should consider investing in the necessary infrastructure and interprofessional staffing (e.g., dedicated physiotherapists, occupational therapists, and respiratory therapists) to reliably implement and sustain early rehabilitation and protocolized sedation weaning. The data suggest that such investments are a rational starting point for building a comprehensive PICS mitigation program.

It is crucial to emphasize that the findings do not support the discontinuation of lower-ranked interventions. The wide credible intervals and low certainty of evidence mean that definitive conclusions about the ineffectiveness of any intervention cannot be drawn. Strategies such as nursing interventions (SUCRA: 53.2%) and enhanced rehabilitation post-discharge (SUCRA: 51.4%) still show a greater than 50% probability of being better than usual care and may address different, equally important aspects of PICS not captured by the mortality outcome. Therefore, a multidisciplinary, multimodal approach remains ideal, and the choice of specific post-discharge interventions should continue to be guided by patient-specific needs, local resource availability, and shared decision-making until more definitive comparative effectiveness data become available.

This review highlights a critical policy implication: the need for a standardized approach to PICS care is undermined by a lack of high-quality evidence, as policymakers and funding bodies should recognize the urgent need to support large-scale, pragmatic, head-to-head randomized trials that can provide the high-certainty evidence required to develop robust, cost-effective, and equitable care pathways for ICU survivors.

Implications for Future Research

This systematic review and NMA summarize the current state of evidence for PICS interventions and illuminate the critical gaps and methodological shortcomings that must be addressed in future research. The findings provide a clear roadmap for the next generation of clinical trials in post-ICU recovery.

The most pressing need is for adequately powered, head-to-head RCTs that compare promising active interventions. The star-shaped network geometry in the analysis highlights the complete absence of direct comparisons between interventions such as early PT/OT, SAT plus SBT protocols, and structured follow-up programs. Future trials should prioritize comparing the interventions that ranked highest in the analysis (e.g., early PT/OT versus SAT plus SBT) to establish their relative superiority directly and with greater precision.

Also, future research must address the clinical and methodological heterogeneity that limited the ability to draw firm conclusions. The components, timing, frequency, and duration of non-pharmacological interventions such as "early mobilization" and "enhanced rehabilitation" must be defined and reported using established guidelines such as the Template for Intervention Description and Replication (TIDieR) checklist, which will improve comparability across studies and facilitate more meaningful meta-analyses. The wide variety of instruments used to measure PICS domains is a major barrier to evidence synthesis. Future trials should adopt a consensus-based core outcome set (COS) for PICS research, including standardized measurement tools and harmonized follow-up time points (e.g., three, six, and 12 months), which will ensure that studies are measuring and reporting outcomes in a consistent manner, enabling more robust future meta-analyses [3].

The high risk of bias in many of the included trials underscores the need for improved trial design and conduct. The blinding of the participants and personnel is challenging for rehabilitation trials, but future studies should make every effort to ensure blinded outcome assessment, particularly for subjective measures such as HRQoL and functional scales. Also, the preregistration of trial protocols with defined primary and secondary outcomes is essential to mitigate the risk of reporting bias.

This review focused on single-modality interventions, but PICS is a multi-domain syndrome that may require multicomponent solutions. Future research should move toward evaluating complex, bundled interventions that target the physical, cognitive, and mental health domains. These trials could employ pragmatic designs, such as cluster-randomized or stepped-wedge trials, to assess the effectiveness of integrated care pathways that span the entire patient journey from the ICU to the community [12,32]. Such studies would provide much-needed evidence on the synergistic effects and cost-effectiveness of a holistic approach to PICS management.

## Conclusions

In this first comprehensive NMA comparing interventions for PICS, no single intervention was found to be superior in reducing mortality among adult ICU survivors. The overall certainty of the evidence is low, but the findings provide a preliminary evidence-based hierarchy, suggesting that in-ICU strategies focusing on combined early physical and occupational therapy and protocolized sedation weaning may offer the highest probability of benefit. The substantial imprecision and heterogeneity across the existing literature underscore an urgent need for future research to prioritize large-scale, methodologically rigorous, head-to-head RCTs. Until such evidence is available, a multicomponent approach, designed to individual patient needs and local resources, remains a pragmatic clinical strategy.

## References

[1] Rawal G, Yadav S, Kumar R (2017). Post-intensive care syndrome: an overview. J Transl Int Med.

[2] Renner C, Jeitziner MM, Albert M (2023). Guideline on multimodal rehabilitation for patients with post-intensive care syndrome. Crit Care.

[3] Needham DM, Davidson J, Cohen H (2012). Improving long-term outcomes after discharge from intensive care unit: report from a stakeholders' conference. Crit Care Med.

[4] Vanhorebeek I, Latronico N, Van den Berghe G (2020). ICU-acquired weakness. Intensive Care Med.

[5] Pandharipande PP, Girard TD, Jackson JC (2013). Long-term cognitive impairment after critical illness. N Engl J Med.

[6] Bienvenu OJ (2019). What do we know about preventing or mitigating postintensive care syndrome?. Crit Care Med.

[7] Jawa NA, Maslove DM, Sibley S (2025). IMPACT-ICU feasibility study: pragmatic mixed-methods randomised controlled trial of a follow-up care intervention for survivors of critical illness and caregivers. BMJ Open.

[8] Schaller SJ, Anstey M, Blobner M (2016). Early, goal-directed mobilisation in the surgical intensive care unit: a randomised controlled trial. Lancet.

[9] Schweickert WD, Pohlman MC, Pohlman AS (2009). Early physical and occupational therapy in mechanically ventilated, critically ill patients: a randomised controlled trial. Lancet.

[10] Herling SF, Greve IE, Vasilevskis EE (2018). Interventions for preventing intensive care unit delirium in adults. Cochrane Database Syst Rev.

[11] Girard TD, Kress JP, Fuchs BD (2008). Efficacy and safety of a paired sedation and ventilator weaning protocol for mechanically ventilated patients in intensive care (awakening and breathing controlled trial): a randomised controlled trial. Lancet.

[12] Friedman D, Grimaldi L, Cariou A (2022). Impact of a postintensive care unit multidisciplinary follow-up on the quality of life (SUIVI-REA): protocol for a multicenter randomized controlled trial. JMIR Res Protoc.

[13] Major ME, Dettling-Ihnenfeldt D, Ramaekers SP, Engelbert RH, van der Schaaf M (2021). Feasibility of a home-based interdisciplinary rehabilitation program for patients with post-intensive care syndrome: the REACH study. Crit Care.

[14] Jones C, Bäckman C, Capuzzo M (2010). Intensive care diaries reduce new onset post traumatic stress disorder following critical illness: a randomised, controlled trial. Crit Care.

[15] Kredentser MS, Blouw M, Marten N (2018). Preventing posttraumatic stress in ICU survivors: a single-center pilot randomized controlled trial of ICU diaries and psychoeducation. Crit Care Med.

[16] Bakhru RN, Flores L, Cain JM (2025). A randomized controlled trial of a post-ICU telehealth care model (WFIT). Am J Respir Crit Care Med.

[17] Lai DJ, Liu Z, Johnston E, Dikomitis L, D'Oliveira T, Shergill S (2024). Exploring the effectiveness of eHealth interventions in treating post intensive care syndrome (PICS) outcomes: a systematic review. Crit Care.

[18] Dong CH, Gao CN, An XH, Li N, Yang L, Li DC, Tan Q (2021). Nocturnal dexmedetomidine alleviates post-intensive care syndrome following cardiac surgery: a prospective randomized controlled clinical trial. BMC Med.

[19] Fuke R, Hifumi T, Kondo Y (2018). Early rehabilitation to prevent postintensive care syndrome in patients with critical illness: a systematic review and meta-analysis. BMJ Open.

[20] Mehlhorn J, Freytag A, Schmidt K (2014). Rehabilitation interventions for postintensive care syndrome: a systematic review. Crit Care Med.

[21] Wade DM, Mouncey PR, Richards-Belle A (2019). Effect of a nurse-led preventive psychological intervention on symptoms of posttraumatic stress disorder among critically ill patients: a randomized clinical trial. JAMA.

[22] Hutton B, Salanti G, Caldwell DM (2015). The PRISMA extension statement for reporting of systematic reviews incorporating network meta-analyses of health care interventions: checklist and explanations. Ann Intern Med.

[23] Page MJ, McKenzie JE, Bossuyt PM (2021). The PRISMA 2020 statement: an updated guideline for reporting systematic reviews. BMJ.

[24] Sterne JA, Savović J, Page MJ (2019). RoB 2: a revised tool for assessing risk of bias in randomised trials. BMJ.

[25] Nikolakopoulou A, Higgins JP, Papakonstantinou T, Chaimani A, Del Giovane C, Egger M, Salanti G (2020). CINeMA: an approach for assessing confidence in the results of a network meta-analysis. PLoS Med.

[26] Khan SH, Perkins AJ, Unverzagt FW (2025). Improving recovery and outcomes every day after the ICU (IMPROVE): a randomized controlled trial. Crit Care Med.

[27] Monard C, Bianchi N, Poli E (2023). Cytokine hemoadsorption with CytoSorb® in post-cardiac arrest syndrome, a pilot randomized controlled trial. Crit Care.

[28] Paulus MC, Kouw IW, Boelens YF, Hermans AJ, Strookappe B, van Zanten AR (2025). Feasibility challenges in protein supplementation research: insights from the convalescence of functional outcomes after intensive care unit stay in a randomised controlled trial. Clin Nutr.

[29] Rood PJ, Zegers M, Ramnarain D (2021). The impact of nursing delirium preventive interventions in the ICU: a multicenter cluster-randomized controlled clinical trial. Am J Respir Crit Care Med.

[30] Walsh TS, Salisbury LG, Merriweather JL (2015). Increased hospital-based physical rehabilitation and information provision after intensive care unit discharge: the RECOVER randomized clinical trial. JAMA Intern Med.

[31] Schofield-Robinson OJ, Lewis SR, Smith AF, McPeake J, Alderson P (2018). Follow-up services for improving long-term outcomes in intensive care unit (ICU) survivors. Cochrane Database Syst Rev.

[32] van Sleeuwen D, van de Laar FA, Simons K (2022). MiCare study, an evaluation of structured, multidisciplinary and personalised post-ICU care on physical and psychological functioning, and quality of life of former ICU patients: a study protocol of a stepped-wedge cluster randomised controlled trial. BMJ Open.

